# The Epigenome Network of Excellence

**DOI:** 10.1371/journal.pbio.0030177

**Published:** 2005-05-17

**Authors:** Asifa Akhtar, Giacomo Cavalli

## Abstract

An initiative funded by the European Union is building a collaborative network of established and younger research groups to tackle key questions in epigenetics.

The term “epigenetics” was first proposed by Conrad Waddington to designate the study of the processes by which the genetic information of an organism, defined as genotype, interacts with the environment in order to produce its observed traits, defined as phenotype [1]. More recently, the term has been used to describe heritable changes in genome function that occur without a change in DNA sequence [2]. These two definitions are closer than they seem. In eukaryotic cells, genomic DNA is packaged by histones and non-histone proteins into a dynamic polymer defined as chromatin. Several enzymes can modify the architecture and the composition of chromatin, both locally and globally, and they can direct the inheritance of local chromatin structures through cell division [3,4]. Thus, an individual's cells all share the same linear sequence of DNA nucleotides—the genome—but different cell types are characterized by the presence of different chromatin flavors of this genome—the epigenomes—that specify the characteristic functions of each cell type and allow the maintenance of the memory of these functions through cell division. Indeed, a large set of genome regulatory processes involve epigenetic determination and inheritance.

Because of the vast complexity of epigenetic regulation, ranging from single genes to the folding of whole chromosomes in the nucleus, epigenetics involves an amazingly wide spectrum of techniques and approaches. These involve structural, molecular, and developmental biology, advanced imaging, genomics, proteomics, bioinformatics, and mathematical analysis. Epigenetic research is applied to different organisms, and it often includes comparative analysis from an evolutionary perspective. Researchers in this field must thus cross borders between different research areas in order to understand the impact of novel findings. This makes epigenetics a highly dynamic area of research, one that at the same time provides an ideal ground for collaborative and synergistic interactions.

With the support of the Sixth Framework Programme of the European Union, a consortium of 25 core laboratories and 26 associate laboratories initiated the Epigenome Network of Excellence (NoE) in 2004. This network will tackle key questions in epigenetic research, integrate young research teams in Europe, provide the scientific community with a large spectrum of resources, including Web-based information, and disseminate scientific knowledge to scientists, students, and the wider public. The Epigenome NoE coordinator, Thomas Jenuwein, says “Epigenetic research represents one of the new frontiers in modern biology with considerable impact on many basic mechanisms and on human biology and disease. With this NoE, the European Commission has allowed the affiliated network members to establish a coherent framework for promoting this exciting research area also to non-members and to interested colleagues outside the field. I am convinced that the enthusiastic spirit of the kick-off meeting at the Mendel Abbey in Brno (September 2004) has inspired many positive and long-lasting signals for a highly functional and integrative network all across Europe.”

The Epigenome NoE has four main aims. The first is to advance scientific discoveries via a strong joint programme of activities, of which a large part is dedicated to research carried out by member labs, alone or in collaboration. These projects range from chromatin modification to cell fate and disease and address some of the “big questions” in epigenetic research (Figure 1).

**Figure 1:**
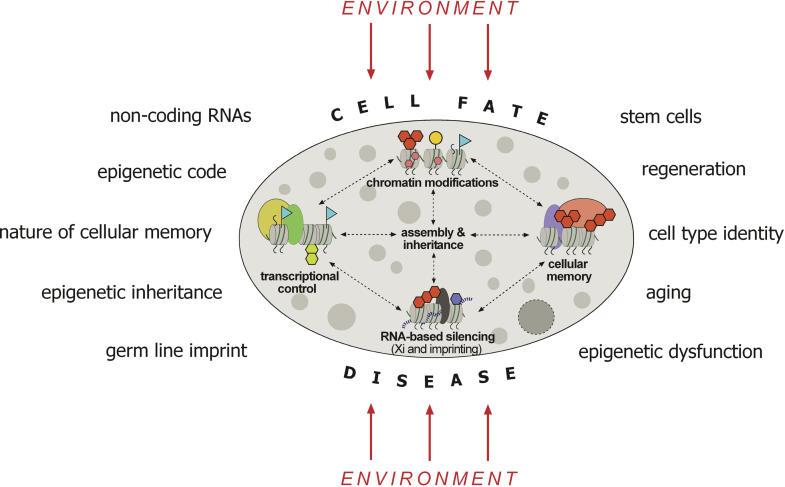
The Impact of Epigenetic Gene Control Diverse biochemical modifications of DNA and histones, such as DNA methylation (indicated by small hexagons), histone methylation (large hexagons), acetylation (triangles), and phosphorylation (circles), occur in response to the environment and modulate chromatin structure. The organization of chromatin controls the access of many proteins, including transcription factors (ovals), to the DNA template and thus regulates gene expression. This epigenetic gene control has an impact on a variety of biological processes, with implications for agriculture and human biology and disease, including our understanding of stem cells, cancer, and aging.

The second aim is to integrate young researchers into the network via a programme called Newly Established Teams (NET). NET provides funding for research and integration in the joint activity programme to promising junior investigators. Newly established researchers often struggle to acquire lab funding, establish collaborations, or simply gather information on available resources in the field. The NET programme provides an ideal platform for such investigators by generating a collaborative atmosphere and opportunities for setting up joint projects and obtaining top-level biochemical and genomic reagents as well as access to state-of-the-art genomic and proteomic facilities. The more established NoE members also act as mentors for the NET participants. In a first wave of integration, twelve NET investigators were selected by an external advisory board (comprising seven United States scientists who are world leaders in epigenetic research) and participated in the kick-off meeting. A second call to integrate ten more NET members will be launched in 2006, advertised towards the end of this year through the Epigenome NoE Web site (http://www.epigenome-noe.net). This call will complete the NET programme for the five-year funding period of this NoE. If successful, this type of integrated effort may serve as an example for future large-scale collaborative projects. The network is gearing up to maintain its funding in the longer term, so that it can continue attracting future generations of top-level young European scientists to epigenetics. Jacques Remacle, EU Commission Scientific Officer, says “To foster research progress in epigenetics, the Epigenome NoE will have to maintain a dynamic structure involving also the participation and integration of newly established research teams. By its flexible nature, the Network of Excellence instrument allows the consortia to evolve over the time to meet new research challenges and/or to promote openness, expansion and durability. The new 12 NET [investigators] that are joining the Epigenome NoE following an open call (57 applications) and a transparent selection process are all of outstanding quality. I strongly believe that their participation and integration to the Epigenome NoE project will benefit epigenetic research in Europe. Following this successful example, the concept of open call for young investigators is now promoted by several NoEs in other research areas.”

A third aim of the network is to provide the scientific community with a large set of resources and information in epigenetic research, and to centralize access to the information already available from existing sources. The key to this aim is the Epigenome NoE Web site (http://www.epigenome-noe.net). During 2005, the site will grow in its role as a database of technology and resources available in individual labs and institutions. Any scientist external to the network will have the chance to establish collaborations with NoE members and thus to access these resources. Furthermore, the Web site is being augmented with searchable databases of experimental protocols, laboratory profiles, and relevant conferences and courses. Interactive Web forums for discussion of each resource and other hot topics in the field of epigenetics will be opened, and job opportunities will be posted regularly. This Web site is therefore expected to become a major source of information for epigenetic researchers.

A fourth aim of the Epigenome NoE is to have annual meetings and workshops that are organized around specific research topics, which will foster collaborations that are likely to be as important, if not more important, than the funding initiative itself. The NoE also aims at reaching the wider scientific community and the public, bringing the concept and the importance of epigenetics to their attention, and raising the level of awareness of the major impact of epigenetic research in important areas such as agriculture and medicine. This will be achieved by organizing public scientific events, by publishing material written in lay language that reviews specific subjects of particular interest, by setting up a Web information service on epigenetics and genomics, and by maintaining an updated “Frequently Asked Questions” Web site. An education page is already posted, which contains a large collection of Web teaching material on issues related to epigenetics. Educational materials developed in house will be added here in the future. The public science component in this NoE should enable us to communicate both the beauty of the underlying scientific questions as well as the significant medical and ethical relevance of epigenetics to the general public, thus bridging a gap between scientists and society. Genetics is becoming deeply rooted in the cultural foundations of modern societies. Epigenetics is equally important, and thanks to the activities of the Epigenome NoE, this relatively new branch of science may soon become familiar to a much wider audience.

## Abbreviations

NET: Newly Established Teams
NoE: Network of Excellence

## References

[pbio-0030177-b1] Waddington CH (1942). The epigenotype. Endeavour.

[pbio-0030177-b2] Holliday R (1994). Epigenetics: An overview. Dev Genet.

[pbio-0030177-b3] Turner BM (1993). Decoding the nucleosome. Cell.

[pbio-0030177-b4] Jenuwein T, Allis CD (2001). Translating the histone code. Science.

